# Ayres Theories of Autism and Sensory Integration Revisited: What Contemporary Neuroscience Has to Say

**DOI:** 10.3390/brainsci9030068

**Published:** 2019-03-21

**Authors:** Emily Kilroy, Lisa Aziz-Zadeh, Sharon Cermak

**Affiliations:** 1 Mrs. T.H. Chan Division of Occupational Science and Occupational Therapy, University Southern California, Los Angeles, CA 90089, USA; lazizzad@usc.edu (L.A.-Z.); cermak@usc.edu (S.C.); 2 Brain and Creativity Institute, University Southern California, Los Angeles, CA 90089, USA

**Keywords:** Autism Spectrum Disorder (ASD), Ayres Sensory Integration (ASI), sensory processing, functional magnetic resonance imaging (fMRI)

## Abstract

Abnormal sensory-based behaviors are a defining feature of autism spectrum disorders (ASD). Dr. A. Jean Ayres was the first occupational therapist to conceptualize Sensory Integration (SI) theories and therapies to address these deficits. Her work was based on neurological knowledge of the 1970’s. Since then, advancements in neuroimaging techniques make it possible to better understand the brain areas that may underlie sensory processing deficits in ASD. In this article, we explore the postulates proposed by Ayres (i.e., registration, modulation, motivation) through current neuroimaging literature. To this end, we review the neural underpinnings of sensory processing and integration in ASD by examining the literature on neurophysiological responses to sensory stimuli in individuals with ASD as well as structural and network organization using a variety of neuroimaging techniques. Many aspects of Ayres’ hypotheses about the nature of the disorder were found to be highly consistent with current literature on sensory processing in children with ASD but there are some discrepancies across various methodological techniques and ASD development. With additional characterization, neurophysiological profiles of sensory processing in ASD may serve as valuable biomarkers for diagnosis and monitoring of therapeutic interventions, such as SI therapy.

## 1. Introduction

**Figure 1:**
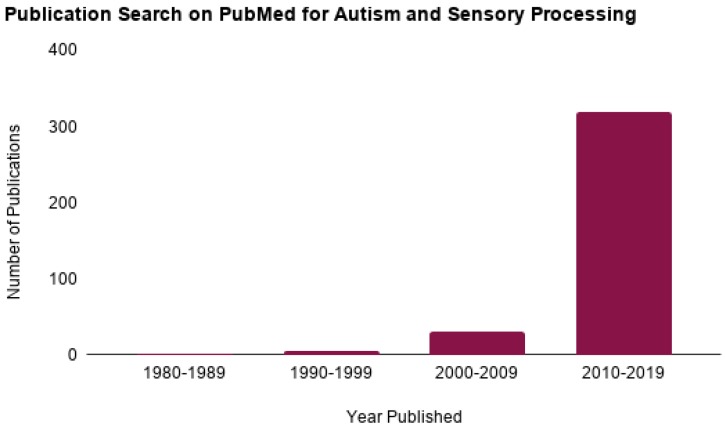
PubMed publication search for Autism and Sensory modalities. Publications by decade for Autism Spectrum Disorder and sensory processing from 1980 through 2019.

Autism Spectrum Disorder (ASD) is a neurological developmental disorder clinically characterized by impairments in social interaction and communication and restricted or repetitive patterns of behavior, interests or activities [1]. In 2018, the Centers for Disease Control and Prevention reported that 1 in every 59 children in the United States has ASD [2]. Among those diagnosed with ASD, it is estimated that over 90% show symptoms of sensory abnormalities [3,4]. Research in ASD sensory processing has become increasingly prevalent since Kanner’s initial description of the condition in 1943 (Figure 1). Recently, in the Diagnostic Manual for Mental Disorders-Fifth Edition (DSM-5), hypo- and hyper-sensory reactivity have been included as diagnostic criteria of ASD [1]. These criteria, however, manifest differently across individuals with ASD; for example, some individuals seem unaware of certain auditory, visual or tactile stimuli (hyposensitive), while others may avoid the same stimuli altogether (hypersensitive). These behaviors can both contribute to and/or overlap with the aforementioned core characteristics and hinder participation in everyday activities [5,6,7].

Over the past 40 years, research on sensory processing in those with and without ASD has enhanced our understanding of how the brain processes sensory input. As early as 1963, Ayres conducted some of the first studies examining sensory problems in a wide range of developmental disorders. Through her work, she conceptualized an intervention approach now referred to as Ayres Sensory Integration (ASI) to treat the sensorimotor foundations of academic skills and other higher order abilities (i.e., planning and organization) [8]. The therapy involves the client interacting with a combination of equipment such as scooters and swings, providing the opportunity to obtain and process enhanced sensory input and develop normal levels of arousal and security when interacting with their environment. Ayres’ intervention approach was founded on the hypothesis that sensory integration (SI) disturbance and other processing abnormalities were, in part, the result of abnormal brain functioning [8]. When Ayres began to study ASD in the late 1970’s, it was considered a rare and little studied developmental disorder (4.5 in 10,000) [9]. Nevertheless, she proposed a theoretical framework to describe the condition. Due to the current prevalence of the disorder, ASD research has significantly increased. In this paper we revisit Ayres’ framework for using a sensory integration approach to address sensory impairment in autism and assess its cogency in light of current neuroscience research.

Much of the technology used today to study brain structure and function was not available to Ayres when she developed her theoretical explanations and interventions. Currently, researchers use methods such as structural and functional Magnetic Resonance Imaging (MRI), eye-tracking systems and electroencephalogram (EEG) to generate information about brain processing. We can now use data collected from these new methodologies to examine their consistency with Ayres’ theories of ASD and consider their implications for SI. The first aim of this paper is to review three aspects of sensory processing deficits in ASD that Ayres discussed in *Sensory Integration and the Child* [8]: registration, modulation and motivation. The second aim is to assess the extent to which current neuroscience research supports Ayres’ postulates.

## 2. Background

Sensory processing involves perceiving, organizing and interpreting information received through sensory systems (e.g., taste, touch, smell, sight, auditory, vestibular) in order to produce an adaptive response. The term “sensory integration” as used by Ayres [8] refers to the ability to produce *appropriate* motor and behavioral responses to stimuli. In her work *Sensory Integration and the Child,* Ayres [8] observed hyper- and hypo-responses to sensory stimuli in individuals with ASD. Specifically, she noted that these individuals exhibited problems in registration (signal detection and interpretation), in modulation (signal inhibition or propagation), in interacting with certain objects, and/or in motivation. Shortly after publishing *Sensory Integration and the Child* [8], Ayres and Tickle [10] investigated sensory disturbances in ASD and their responses to SI therapy specifically. In this retrospective study, the authors found that individuals with hyper-reactivity (a disorder of modulation) had better outcomes than those who were hypo-reactive and proposed that children who register sensory input respond better to therapy than those who do not [10]. Although Ayres did not identify the neural structures that underlie these disturbances in her publications, she implicated two neural systems in registration and modulation: 1) the limbic system and 2) the vestibular and proprioceptive systems. Her manuscript with Tickle [10] and the chapter on Autism in *Sensory Integration and the Child* are the two main publications in which Ayres delineates her views of SI and ASD. The theory that disruptions in the limbic system also contribute to motivation deficits in ASD is further found in unpublished archival documentation of Ayres’ work and lectures preserved at the University of Southern California (USC) library archives [11].

Components of impaired sensory integration in ASD according to Ayres.

Registration: Registration is the detection of sensory sensations within the central nervous system. Ayres [8] used the term “registration of sensory information” which expands beyond the clinical definition of the initial detection of a stimulus to also include the recognition of significant meaning of sensory stimulation. Ayres [8] suggested that some children with ASD do not register sensory inputs properly; and as a result, these children allocate attention differently from typically developing (TD) children. For example, children with ASD may not register the presence of a salient stimulus (e.g., a person walking in the room, the appearance of a new toy or the sensation of a puff of air on their necks) the way typical children do. Ayres [8] hypothesized that registration problems are located in the limbic system (also known as emotion-related brain regions), which she described as responsible for ‘deciding’ what is brought to consciousness and whether we will act on it (pp. 124–125). Ayres also identified the vestibular nuclei as being involved in the registration of visual input and making it “meaningful” to the child (p. 125).

Modulation: Modulation is the ability of the brain to regulate inhibition or propagation of neural signaling. Sensory modulation reflects adjustments made in response to continual physiological processes to ensure adaptation to new or changing sensory information. Ayres [8] defined modulation as the “brain’s regulation of its own activity” (p. 182). She proposed that children with ASD not only fail to register sensory input properly but also have trouble modulating input that they do register. She suggested that over- or under-activity, especially in response to vestibular and tactile sensations, may manifest in gravitational insecurity (fear of movement, especially when not in the upright position), tactile defensiveness (fight, fright or flight reaction to light touch that most others would consider non-noxious) or a combination of both.

Motivation: Ayres [8] described motivation as the desire or willingness to respond to a stimulus that has been registered or to ignore it (pp. 127–128) and proposed that children with ASD have a motivation deficit in children with ASD. More specifically, she observed that individuals with ASD may have limited interest in doing purposeful or constructive activities. According to Ayres, although children with ASD have the motor ability, they may not have enough motivation to actually carry out certain activities. Ayres located this problem in “the ‘I want to do it’ function of the brain” (p. 127) but she did not attribute it to a particular brain region in her published work. Lectures and notes written by Ayres preserved at the USC library archives, however, indicate that Ayres’ implicated the amygdala—a subcortical region of the limbic system—for this “do something” function. Ayres additionally identified poor “inner drive” and environment/body precepts as contributing to motivation impairments but did not delineate how these components interacted. Henceforth, a lack of motivation will be referred to as motivation deficit, which is consistent with other researchers’ definitions of motivation [12,13].

Given the research tools available at the time, Ayres [8] established this basic framework for conceptualizing sensory processing deficits in registration, modulation and motivation in children with ASD. To engage in an updated discussion of her conceptualizations, this paper examines current neuroscience research related to the aforementioned postulations, including studies investigating emotion-related brain regions, neural responses to sensory stimuli and value of stimuli in individuals with ASD compared to TD individuals.

## 3. Current Neuroscience Evidence

### 3.1. Registration and Modulation: Emotion-Related Brain Regions (Previously Referred to as the Limbic System)

As previously stated, Ayres [8] suggested that a collective set of brain regions associated with emotions, often called the limbic system, is responsible, in part, for sensory registration. She posited that these regions of the brain are atypical in children with ASD and therefore, these children do not register and value stimuli in the same way as TD children. Moreover, Ayres proposed that, “the more poorly this part (of the brain) is working, the less the autistic child will respond to therapy” (p. 124). In other words, the more abnormal the limbic system (she did not specify functional or structural differences), the less likely SI therapy will effectively ameliorate sensory processing impairments. Within the last decade, current neuroscience research has affirmed Ayres’ assertions that limbic emotion-processing regions are impaired in individuals with ASD [14,15,16,17,18]. Findings from this research provides evidence to support predictions of impairments of registration, as well as modulation of sensory processing in ASD. However, to date, there is no research that specifically tests Ayres theories regarding effectiveness of interventions using a SI approach as a function of neural functioning in ASD. For a review of behavioral outcomes of ASI therapy see [19,20,21,22].

The term, limbic system, refers to a specific set of regions thought to encompass emotion-related brain regions. The system is comprised of several subcortical nuclei and cortical structures including the insula, hypothalamus, hippocampus, parahippocampal gyrus, amygdala, fornix, mammillary body, septal nuclei, cingulate gyrus and dentate gyrus on both sides of thalamus [23]. These regions are involved in emotion, motivation, learning, memory and certain aspects of sensory processing. Additional regions outside of the limbic system, such as the prefrontal cortices and ventral and medial sectors, are now known to also be important to emotion processing [24]. Here we will collectively refer to these regions as emotion-related brain regions, a term that is currently preferred [25].

#### 3.1.1. Brain Structure and Function

Consistent with Ayres’ theory, current research has thoroughly documented that individuals with ASD have both structurally and functionally atypical emotion-related brain regions [17,18,26,27,28]. These regions have been observed to be abnormal in size and function across the lifespan [29], however, it is still not clear if volume or responsivity are increased or decreased or how these abnormalities underlie symptomology in ASD. Structurally, volume size trajectories across development for these regions start out larger in children with ASD than in TD children [30,31]. It is thought that children with ASD have an overgrowth of neurons that diminishes in adolescents [32]. In older adolescence and adulthood, there are mixed findings regarding emotion-related structures. For example, it has been observed that adults with ASD have reduced amygdala [33] and hippocampus volume compared to typical individuals [34,35,36] while other studies have reported increased hippocampal volume [37] or no differences [38]. In some instances, the cortical and subcortical volume of regions in this system have been related to cognitive functioning (i.e., social processing, attention) [39,40,41]. In general, increased volume in structures has been found to be related to ASD deficits [42]. However, it is not well understood how structural components of this system relate to sensory processing specifically. A few white matter studies have reported variation in tracts connecting emotion-related brain regions to auditory and cognitive processes [43,44] and white matter volume related to motor skills [45]. Motor impairments in ASD have also been found to be positively correlated with white matter volume in regions of the brain stem, the central tegmental tract/medial lemniscus [46]. The lemniscus conveys tactile information and proprioception to the cortex via the thalamus. This finding provides some support for Ayres theory that the brainstem structures are related to sensory motor impairment in ASD. However, more research is needed to fully understand the specificity of how brain structures are related to sensory impairments in ASD.

Studies of neural activation (function) in emotion-related brain regions have similarly reported abnormal functioning in ASD. The amygdala in particular has been consistently found to be dysfunctional. Neuroscience research published at the time of Ayres’ career reported that the amygdalae contain a larger proportion of neurons that signal valance than other emotional-related brain regions and are responsive to motivationally significant stimuli [47]. Ayres predictions about the connection between impaired detection and understanding of the meaning of stimuli (registration) and the “limbic system” may indeed, in part, be due to functional disruptions in the amygdalae in ASD. In Ayres’s lectures and notes, she identified the amygdala as being involved in sensory registration (USC Archives) [11]. The amygdala has since continued to be implicated in recognizing valence in stimuli [48] as well as encoding reward associations of visual stimuli and attention [49]. Moreover, many studies report attenuated amygdala activity when individuals with ASD perform social tasks compared to typical individuals [50,51,52]. Yet, other researchers have found that in individuals with ASD that the amygdala over-activates with eye-gaze compared to typical controls and that in these individuals amygdala activation is correlated to time spent gazing (i.e., looking at eyes) [53,54].

Hyperactivation in the amygdala to eye contact may indicate a modulation dysfunction in ASD in which some have proposed an indication for why individuals with ASD avoid eye contact with others [54,55]. This hypothesis suggests that avoiding eye contact is a motivational response [56], which aligns with Ayres’ understanding of motivation (see Section 3.2). Alternatively, it also has been posited that individuals with ASD are not aversive to eye-gaze but are indifferent to it; that is, they do not perceive others’ eyes as informative or salient stimuli [57,58,59]. This lack of saliency detection may be why other studies have observed amygdala hypoactivation. In regard to “registration,” Ayres discussed individuals with ASD as having impairments in both the detection of a stimulus at the level of the central nervous system (CNS) and in salience perception. Current findings support both definitions. The amygdala has been frequently implicated in attention [49] and recognizing valence in stimuli [48] as well as in encoding reward associations of visual stimuli [49]. In addition to “limbic regions”, Ayres specifically hypothesized that the vestibular nuclei (located in the brain stem) were involved in registering visual input and in helping to make it meaningful to the child [8] (p. 125). To date, no neuroimaging studies have investigated the vestibular nuclei and “registration” in ASD specifically, however, it is possible that abnormalities in the amygdala—which receives projections from the medial vestibular nucleus via autonomic nuclei and parabrachial nucleus [60]—may play a role in registration disturbances.

The insula is an important emotion-related region not identified by Ayres that is involved in registration deficits in ASD [61]. The insula is important for attention and is a core node of the salience network, which responds to novel and relevant sensory stimuli and is important for cognitive control and switching between default mode networks (introspective functions) to task-based networks [62]. The insula also acts as an integration center for physiological and emotion perception [63]. This cortical region has been repeatedly reported to be altered in ASD [64,65,66]. Insula abnormalities have been observed in individuals with ASD while performing various cognitive tasks, such as social processing tasks, emotion processing, spatial attention [67,68], set-shifting tasks [69] and executive function tasks [64,70,71].

Together, current neuroimaging research supports Ayres’ framework by providing evidence that emotion-related brain regions are structurally and functionally different in individuals with ASD compared to TD individuals. Further, research findings suggest that these regions are important for registration of sensory information and that they are disrupted in a way that impairs sensory modulation. These differences, however, are multifaceted and may depend upon methodology (i.e., age, stimuli, instruction, etc.). Individual regions, such as the amygdala or insula, are part of a much larger emotional processing network and are physically and functionally connected to other regions. The connectivity between these regions and other brain regions also contribute to SI disruptions in ASD and are discussed below. Functional task-based MRI studies that directly investigate how different sensory experiences are processed in ASD are reviewed later.

#### 3.1.2. Functional Connectivity

While characterization of brain regions provide insight into ASD dysfunction, a systems level approach in the last decade has provided additional understanding into how sensory information is communicated within and between networks. Recent research has examined network functioning of specific regions, including emotion-related brain regions in those who are TD and individuals with ASD. Although there are some inconsistencies [72], significant evidence to indicates that individuals with ASD have atypical network connectivity. This has led some to characterize ASD as a disorder of altered brain connectivity [73,74]. These functional connectivity studies identify areas of the brain where activation patterns are synchronized across time. For example, Rudie et al. [75] observed that children and adolescents with ASD displayed reduced functional connectivity between the amygdala and secondary visual areas while passively viewing emotional expressions compared to the TD control group. Moreover, the study found that increased ASD symptom severity correlated with decreased connectivity, thereby suggesting that reduced communication between visual input and emotion responsivity is related to ASD symptomatology. These and similar findings [73,76] also lend support to Ayres’ postulation regarding the severity of abnormal “limbic” functioning and SI by demonstrating that the degree of symptom severity corresponds with the degree of sensory registration disruptions. Indeed, Ayres did predict that deficits in sensory processing hinder motor planning, which may ultimately result in trouble with more complex behavior, including social and emotional cognition [8] (p. 129). How this network responds to sensory stimulation is discussed later.

In addition to task-based connectivity networks, several functional networks are intrinsic to neural functioning and are thought to be related to different aspects of sensory processing (i.e., default mode network, salience network, motor network, auditory network, etc.). These networks closely resemble functional networks that are active when performing tasks [77,78] and are often referred to as resting state networks. Findings from current resting state studies suggest that individuals with ASD have disrupted connectivity in many of these networks. Hypo- or hyper-connectivity findings suggest that individuals with ASD have greater and/or weaker communication with networks compared to typical peers, regardless of specific tasks or stimuli. Several of the studies correlate behavioral symptomatology to the strength of these network connections, indicating that impairments in neural modulation are not restricted to region specific impairments. In a recent study Maximo and Kana [79] reported that individuals with ASD demonstrated network dysfunction in many sensory processing networks, including hyperconnectivity in auditory-subcortical, motor-thalamic and lateral visual-basal ganglia networks and hypoconnectivity in medial visual-subcortical networks. This and other studies indicate that functional networks supporting primary sensory processing are impaired in ASD at the intrinsic level [15,79,80,81,82,83] and may prevent appropriate integration of sensory information in higher order sensory integration regions. Ayres [8] described individual’s impairments in registration as “capricious” due to neural inefficiencies. Indeed, findings of reduced network efficiency are observed in other types of network analysis, such as graph theory analysis [51,84].

#### 3.1.3. Neural Responses to Aversive or Pleasant Sensory Stimulation

An increasing number of neuroimaging studies examine neural processing during exposure to specific kinds of sensory input (i.e., tactile, auditory, visual) [85,86]. These studies demonstrate that individuals with ASD have differences in “limbic system” responsivity to visual input (flashing checkerboard), auditory input (loud noises) and tactile input (aversive or pleasant material) compared to TD participants [87,88,89].

Cascio and colleagues [89] found tactile pleasantness (determined by a −100 to 100 hedonic rating scale) varied in ASD compared to TD adults. Although psychophysical or perceived ratings of roughness and pleasantness were largely similar across the two groups, the ASD group gave pleasant (i.e., cosmetic brush) and unpleasant (i.e., plastic mesh) textures more extreme ratings than did controls. Moreover, ASD participants rated neutral textures with more variance than those in the control group, thus indicating that ASD adults are less consistent when evaluating ambiguous stimulus. It is possible that this variance in rating reflects the degree of sensitivity and discriminative ability that some individuals with ASD may have. Further, changes in blood oxygenation level-dependent (BOLD) signal in response to stimulation differed substantially between the groups; the ASD group exhibited diminished responses in the posterior cingulate cortex and the insula, particularly for pleasant and neutral textures, compared to the control group. For the most unpleasant textured stimuli, the ASD group exhibited greater BOLD response than controls in affective somatosensory processing areas, including the posterior cingulate cortex and the insula. The insula’s amplitude of response to the unpleasant texture was positively correlated with social impairment as measured by the Autism Diagnostic Interview-Revised (ADI-R [90]). According to these results, individuals with ASD show diminished response to pleasant and neutral stimuli and exaggerated emotion-related region responses to unpleasant stimuli, providing support for Ayres’ [8] theory of modulation deficits in ASD, both behaviorally and neurologically.

In a set of complementary studies, high-functioning youth with ASD and age- and Intelligence Quotient-equivalent TD youth were presented with adverse sensory stimuli during an functional magnetic resonance imaging (fMRI) scan [86,91,92,93]. In response to the stimuli, ASD participants displayed greater BOLD activation in primary sensory cortical areas and limbic structures including the amygdala, hippocampus and orbital-frontal cortex [92]. In both groups, the level of activity in these areas positively correlated with parent’s ratings of their child’s sensory responsivity on the Sensory Over-Responsivity Scales (SOR) [92,94]. In a more recent study investigating functional connectivity between emotion and sensory brain regions during sensory aversion of auditory and tactile stimuli (white noise and scratchy texture), Green and colleagues [91] found that individuals with ASD displayed aberrant modulation of connectivity between the thalamus (pulvinar nucleus) and sensory-motor regions compared to their TD peers. Specifically, they posited that increased amygdala-pulvinar connectivity may be related to selective attention in ASD since the amygdala signals the brain to attend to distracting sensory stimuli. Taken together, these results demonstrate that youth with ASD show neural hyper-responsivity to adverse sensory stimuli and that behavioral symptoms characterized by SOR may be related to both heightened responsivity in primary sensory brain regions and emotion-related brain regions at both the regional and network level. Again, these findings provide support for Ayres theory of disrupted modulation of sensory stimuli in emotion-related regions in ASD.

In a follow up study, Green and colleagues [86] investigated the effect of adverse sensory distractions on the brain while participants performed a social cognition task involving the interpretation of visual and audio cues (sarcasm task [95]). When the aversive stimuli (scratchy material) was applied while participants completed the task, individuals with ASD showed decreased activations in language and dorsolateral and dorsomedial prefrontal cortex compared to when no aversive stimulus was applied. Activation in these regions was not correlated with SOR scores, suggesting that the reduced activation was not related to sensory deficits. When the participants were given instructions to focus on the face and voice during the social task, the addition of the adverse stimulus did not decrease activation in the ASD group but increased activation in the medial prefrontal cortex. This finding demonstrates that explicit instructions for attention can increase medial prefrontal activation during this sarcasm task. The researchers of this study theorize that sensory stimuli can disrupt neural networks processing social information in ASD and that instruction may mitigate this effect. All of the fMRI sensory studies described above corroborate Ayres’ [8] postulate that emotion-related brain regions have atypical modulation responses when experiencing different sensory modalities in children with ASD compared to TD individuals.

### 3.2. Motivation: Attraction and Reward in the “I Want to Do It Part of the Brain”

Conscious registration of sensory stimuli results in one of two reactions: to respond to the stimuli or to deliberately ignore it (unconscious registration will not be discussed here, for reviews see Fang, Li, Chen, and Yang [96] and Morris, Öhman, and Dolan [97]. Ayres [8] suggested that a part of the brain that motivates a response has an “energizing effect” that initiates behavior when encountering a stimulus and triggers the desire to do something new or different (p.127). She postulated that the part of the brain that decides, “I want to do something” is impaired in ASD and proposed that children with ASD do not receive the same pleasure or get the same reward from activities as do TD children when they register sensory stimuli. As such, they are not motivated to engage in the activities. Ayres attributed an ASD child’s lack of acknowledgement or reward of important stimuli, in part, to the child’s inability to register and/or to perceive the meaning or potential of things otherwise considered meaningful by others. Ayres stated that children with ASD did not understand the meaning of stimuli through observation alone; instead, they learned best through experiences and that ASI therapy works with individuals to incentivize the registration of sensory sensations. In other words, Ayres postulated that children with ASD do not generalize meaning from one sensory stimulus to another which hinders their drive to do things. However, rewarding the child could help the child to register the stimuli and motivate them engage with it. She gave an example of a child who knew how to ride a tricycle but did not understand the meaning of riding a scooter (that it functions similar to the tricycle and is something to ride) and therefore, was not motivated to try it [8] (p. 128). Ayres stated that simply registering the image well enough to notice or pay attention to it is not always sufficient to motivate the child’s inner drive to interact with it. Ayres suggested that the inner drive malfunctions in individuals with ASD but did not state how. Simultaneously, Ayres acknowledged that some children with ASD sought out and experienced much pleasure from self-selected sensory input. Therefore, a child with ASD may be unmotivated by some stimuli because he or she is unable to imagine the ways in which engagement with these stimuli would be satisfying or rewarding. Ayres hypothesized that this poor understanding of the meaning of a stimulus was the result of poor ability to think abstractly as well as poor environmental and bodily precept. Ayres described having a bodily precept (schema) as being an important part of motor planning and motivation for purposeful activity.

**Figure 2:**
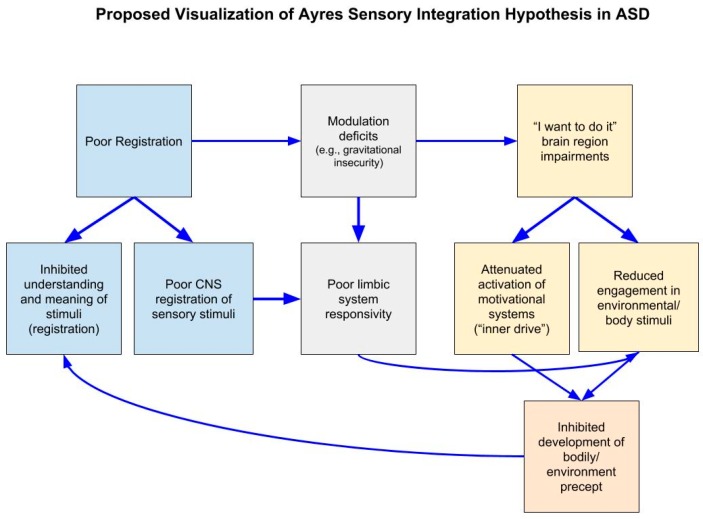
A proposed visualization of Ayres Sensory Integration Hypothesis in autism spectrum disorders (ASD). CNS: Central nervous system.

Ayres [8] outlined how multiple sensory processing impairments, including motivation deficits, hinder motor development in ASD. She hypothesized that the lack of motivation to engage with a stimulus inhibits the development of a precept which contributes to impaired understanding of the potential meaning of the stimulus (poor registration), which also perpetuates the lack further motivation to engage. This is in addition to other hindrances, such as modulation deficits, that motivate a child to not engage with a stimulus (see Figure 2). While the specific motivation mechanisms that Ayres proposed have not been tested (a relationships between bodily percept and motivation, “inner drive” and “I want to do it” impairment), our current understanding of neural functioning suggest that multiple brain regions and neural networks are responsible for motivating a response—in Ayres own words—to “do it,” such as the reward system and the cerebellum [98,99]. These systems have been tested and found to be disrupted in ASD as described later.

Over the last few decades one prominent theory that has come forth and aligns with Ayres’ understanding of impaired motivation in ASD is the social motivational theory of autism [13,100]. This theory posits that individuals with ASD are not rewarded by social stimuli as are TD individuals. In particular, studies have documented that children with ASD have reduced motivation for social rewards, such as faces and human voices, compared to typical peers [13,101]. For example, one study found that the sound of the human voice was less pleasing or rewarding to children with ASD than other noises [102]. At the neurological level, in an fMRI study involving individuals with ASD, results indicated reduced connectivity between the neural structures that process the human voice and the neural regions that process reward [103]. Other researchers have reported similar findings utilizing other social visual stimuli, such as faces and biological motion [104,105,106,107].

An important component of motivation and reward in both ASD and TD individuals is attraction. Attraction can be defined as the action or power of evoking interest, pleasure or liking for someone or something and can be measured through various signals or biomarkers [108,109,110]. One primary behavioral biomarker is the amount of time an individual spends looking at an item, person or space. In a lecture by Ayres given in 1981, she explained that she had not yet found a reliable procedure to measure eye gaze (USC archives) [11]. Since that time, neuroimaging techniques in conjunction with sophisticated eye-tracking studies have quantitatively shown that children with ASD are attracted to different stimuli than TD children [13,111,112]. It has been well established that individuals with ASD focus more on non-socially relevant information when scanning a face (i.e., mouth, nose) than do TD individuals. As previously discussed, individuals with ASD look significantly less at core features of the face such as the eyes [113]. In addition to abnormal amygdala responses indicating registration and/or modulation deficits that may result in individuals with ASD being motivated to avoid eye gaze, this lack of motivation has also been attributed to a decreased reward value for social stimuli. Reward system deficits in individuals with ASD have been observed when viewing eyes [109], as well as other social stimuli, including faces and biological motion [101,106,107,114,115,116,117].

Attenuated attraction and reward for various social stimuli in ASD has been observed across development beginning in early infancy. Klin et al. [118] found that infants with autism failed to recognize displays of biological motion in the form of a point light display technique—a projection reflecting natural biological motion by limited points of light that produce the visual phenomena of a moving animate object (i.e., walking). On the other hand, children with ASD were highly sensitive to the presence of a non-social, physical contingency that occurred within the stimuli presented by chance [118]. These results empirically support Ayres supposition that children with ASD are not attracted/have preference for stimuli that typical children find meaningful and have difficulty generalizing meaningful information. While the above eye tracking studies demonstrate reduced motivation for social stimuli, they do not elucidate the neurological underpinnings of this disruption. In a more recent study, neural activity elicited in social motivation/reward regions (orbitofrontal cortex, putamen, ventral striatum) while viewing biological motion point light display predicted outcome in pivotal response treatment [119] in children with ASD [120]. This study is the first of its kind to provide evidence that neural signatures in brain circuits implicated in social information processing and social motivation/reward can predict treatment effectiveness at the individual level in children with ASD.

Disturbances in motivation, especially social motivation, can be detrimental to social development and participation in meaningful occupations across the lifespan. Ayres [8] suggested that this reduced interest and salience of stimuli results in an underdeveloped precept and is the reason why individuals with ASD are not motivated to “do things”. Ayres thought that this lack of reward leads to reduced motivation, which she described as an important factor in ASD-related behavioral deficits. Although the regions that Ayres had in mind when she wrote about the “’I want to do it’” part of the brain are uncertain, motivational and reward systems are now better understood. Functionally, reward processing also involves cortical activity in the anterior cingulate cortex, orbitofrontal cortex and ventral striatum [121,122]. These areas, as well as other reward-related regions such as the insula [123,124], underlie reward processing in humans for food rewards [125], monetary rewards [126,127] and social rewards (e.g., viewing faces) [128]. Individuals with ASD have reduced activation in these regions for both social and monetary rewards [101,106]. There is also evidence to suggest that abnormal motivation in ASD extends to primary rewards, such as food. In a study by Cascio [89] researchers asked children with ASD and typical peers to fast for four hours before they viewed images of high-calorie foods. The researchers observed that the ASD group had a stronger response to food cues in reward areas (bilateral insula along the anterior-posterior gradient and in the anterior cingulate cortex) compared to the TD group.

Another core brain system for processing reward value is the mesolimbic reward pathway. This pathway connects the ventral tegmental area (VTA) and the nucleus accumbens (NAC) [129] and is involved in evaluating, regulating and reinforcing appetitive behaviors through dopaminergic signaling [130]. This pathway is important for detecting and modulating responses to rewarding stimuli and modulating seeking behaviors [131]. Reduced functional and structural integrity of the mesolimbic pathway has been reported in individuals with ASD and related to parent-report measures of social interactions [132]. Therefore, functional and structural abnormalities in the reward systems of individuals with ASD may account for their lack of reward, reinforcement of behaviors and subsequently reduced “energizing effect” that Ayres [8] described.

In addition to the reward system, other brain regions help drive and reinforce seeking behavior which also may be consistent with the “energizing effects” Ayres [8] described. The lack of motivation to explore and seek out novelties implicates atypical cerebellum processing in ASD. Pierce and Courchesne [133] directly linked the likelihood of individuals with ASD to explore novel stimuli to the magnitude of cerebellar hypoplasia of vermis lobules VI–VII. Other non-clinical experimental brain imaging studies that have investigated the relationship between the cerebellum and motivation have reported evidence that suggesting links between motivation, emotion and action and connections with emotion-related brain regions [134,135]. The cerebellum possesses several somatotopic maps of the body and connects to reward and emotion related regions such as the amygdala and ventral tegmental area [136] via the fastigial nuclei of the deep cerebellar nuclei providing evidence that the cerebellum is involved in motivation and emotion [135]. Some models of the cerebellum propose that it monitors emotion related brain regions and provides feedback that directs behavior [137,138]. Likewise, functional and structural imaging studies have found neuroanatomical abnormalities in the cerebellum in individuals with ASD [139,140]. Taken together, this growing body of research provides evidence consistent with Ayres’ [8] postulate that individuals with ASD have deficits in motivation that result from abnormalities in the brain. Moreover, they also provide evidence that “limbic” structures, such as the amygdala and insula, are related to motivation deficits observed in ASD.

## 4. Discussion

This paper revisits Ayres’ [8] primary postulates regarding sensory processing deficits in registration, modulation and motivation in individuals with ASD and examines them in light of current neuroscience research. To this end, we reviewed studies of sensory processing and sensory integration that used a variety of modern neuroimaging technologies and techniques to examine components related to sensory processing in ASD. Findings from these studies provide preliminary evidence to support Ayres’ postulates and expand upon her original theories of sensory processing in individuals with ASD.

While registration has not explicitly been tested at the neurological level, Ayres’ [8] framework is corroborated by findings of abnormal structure and function of regions important for identifying relevant information and reduced eye-gaze to salient information in ASD. The emotion-related brain regions implicated in her theories of registration are commonly found to be disrupted in this group across multiple levels of neurobiology (structure, function, network organization). Emotion-related regions such as the amygdala and insula play important roles in identifying salient information; in ASD these regions respond abnormally when attending to relevant social and sensory information [50,52,75,93]. Reduced activation to primary sensory (e.g., touch, sounds) and secondary sensory (socially relevant) stimuli in these regions support Ayres’ theories that they are not detecting the information in the same way as TD peers. This disruption contributes to impairments in network functioning as well. Connectivity networks involving these regions also are altered in ASD [15,79]. Current resting state findings suggest that individuals with ASD, in general, have less efficient network connectivity and trouble switching from passive internal thoughts to functional tasks [66,141]. Overall, research confirms that registration functions involved in the detection of stimuli and in the understanding of the stimulus meaning are impaired in ASD.

In addition to “limbic” regions, Ayres’ hypothesized that the vestibular nuclei are involved in sensory registration. In the 1970’s, several important papers were published implicating the vestibular system in ASD [142,143,144,145]. To our knowledge, no significant research published to date uses neuroimaging techniques to directly investigate vestibular processing in ASD. Main factors contributing to the paucity of neuroimaging research related to vestibular functions is twofold: (1) current MRI techniques are highly sensitive to motion, which distorts the rendering process and introduces artifacts in the data; and, (2) the vestibular nuclei are located in the brainstem, which is notoriously difficult to collect quality data from due to motion artifacts produced by blood boluses. Nevertheless, a few recent studies have indirectly investigated vestibular pathways and have implicated components of vestibular processing in ASD, such as impaired thalamus functioning [146,147,148,149]). Non-imaging studies have continued to examine vestibular function behaviorally and have found that atypical responses of the rotational vestibulo-ocular reflex (rVOR; which functions to maintain stable vision during head movements) indicating alterations in cerebellar and brainstem circuitry [150]. Others, however, reported typical rVOR function when measuring the head tilt-suppression mechanism of rVOR [151].

Research providing evidence for registration impairments also provides evidence for modulation deficits in ASD. Hypo- and hyper-responsiveness to sensory stimuli (now listed as diagnostic criteria in DSM-5 [152] and its correlation with sensory responsivity supports Ayres’ second postulate about abnormal modulation of sensory input in ASD. Ayres [8] hypothesized that poor sensory stimulus registration and modulation impairments were closely linked. While some eye-tracking research submits that individuals with ASD do not register certain visual stimuli (i.e., eye contact), increased activation in the amygdala when gazing at the eyes indicates modulation problems as well. Current imaging research has demonstrated that many emotion-related brain regions do not respond to sensory stimulation in the same way in ASD compared to TD individuals [85,86,89,91,92]. Moreover, in the ASD groups, correlations of SOR scores with activity elicited during aversive stimuli experiences further indicate that individuals with ASD have abnormal neural responses as a function of their sensory impairments. Again, these differences may be due to variation and abnormality in both structural and functional activity and connectivity. According to a meta-analysis, which reviewed fourteen distinct studies, individuals with ASD demonstrated both under- and over-responsivity to sensory stimuli [153].

Ayres’ third postulate regarding motivation, is her least cultivated. Ayres provides little theory regarding the neurological mechanisms that are involved in “wanting to do something”. However, she did describe several symptoms in individuals with ASD that may contribute to their reluctance to respond to sensory stimuli or to do new and different things (Figure 2). These components include malfunctions in the “I want to do it” part of the brain and inner drive, an individual’s inability to have abstract thought and register meaning of sensory stimuli, modulation impairments motivating aversion to stimuli and reduced quality of environment and body precept. Several theories and neural mechanisms have since been implicated and align with Ayres’ proposed components. The social motivation theory [100] is congruent with Ayres statements that children with ASD do not register the potential meaning of things and are, therefore, not inclined to “do” anything in response. Imaging research on social reward processing has demonstrated that children with ASD do not recruit the same reward processing regions for socially salient stimuli, as well as other rewards (monetary, food) compared to TD children [101,107,125]. The theory attributes reduced attraction to social stimuli to a lack of reward system activation. It is also possible that these same social reward processes are linked to reward processing abnormalities implicated in sensory processing in general. Functional connectivity research findings demonstrate an overall reduction in primary sensory regions and reward system connectivity in ASD [154,155]. Studies using sensory stimuli as a reward would provide additional data to support this theory. It is still important to clarify whether individuals are unmotivated by sensory sensations or find it aversive. Given the heterogeneity in ASD symptomatology, this may vary across individuals. Furthermore, cognitive abilities, such as abstract thinking and intelligence, must be considered because deficits in motivation and reward may be due to poor understanding of the meaning of verbal and visual commands or intent, as Ayres suggested. This may be too difficult to investigate because eligibility to participate in neuroimaging studies is typically restricted to those with an IQ over 80 in order to ensure participant safety and data quality, which poses a particular limitation on studies involving persons with ASD. Finally, the cortical and subcortical connections within the reward network and with the cerebellum are promising directions for future neuroimaging research. While Ayres did not name the brain region responsible for “wanting to do it”, research on reward circuitry and the cerebellum is warranted given their respective roles in motivation, as well as the reported structural and functional abnormalities of this region in ASD [156]. Exploring the connections between these regions may help elucidate specific neural circuits and sensory pathways that are impaired in individuals with ASD.

## 5. Conclusions

With the advancement of neuroimaging and other innovative technologies, scientists have begun to map the structure and function of the brain areas that may underlie sensory processing deficits in ASD. Ayres’ predictions about sensory registration, modulation and motivation are strongly supported by the findings of various studies. Ayres observed sensory heterogeneity in ASD and predicted that it would have implications for therapy. She theorized from her own work that individuals with modulation but not registration deficits would respond better to SI therapy. Stratifying sensory phenotypes of ASD with neurological markers may lead to improved individualized therapy. However, today very little research has linked ASD neurosignatures to therapeutic outcomes. Further research is necessary to better understand the relationship between neural abnormalities in ASD and therapeutic approaches intended to ameliorate sensory impairment symptoms and to promote easier participation in everyday life activities. To our knowledge, no published studies have specifically investigated the neural response to Ayres sensory integration therapy in individuals with ASD. Research is needed to examine whether intervention using a sensory integration approach will help improve sensory registration and/or modulation impairments in ASD by developing a more efficient network connectivity.
